# Lipid metabolism in tumor microenvironment: novel therapeutic targets

**DOI:** 10.1186/s12935-022-02645-4

**Published:** 2022-07-05

**Authors:** Xingkai Liu, Ping Zhang, Jing Xu, Guoyue Lv, Yan Li

**Affiliations:** 1 Department of Hepatobiliary and Pancreas Surgery, General Surgery Center, The First Hospital of Jilin University, No. 71. Xinmin Street, Changchun, 130021 Jilin China; 2 Department of Echocadiography, The First Hospital of Jilin University, No. 71. Xinmin Street, Changchun, 130021 Jilin China; 3 Department of Surgery, School of Medicine, University of Louisville, 511 S Floyd ST MDR Bldg Rm324, Louisville, KY 40202 USA

**Keywords:** Lipid, Fatty acid, Cholesterol, Lipid metabolism, Tumor microenvironment

## Abstract

Bioactive lipid molecules have been proposed to play important roles linking obesity/metabolic syndrome and cancers. Studies reveal that aberrant lipid metabolic signaling can reprogram cancer cells and non-cancer cells in the tumor microenvironment, contributing to cancer initiation, progression, metastasis, recurrence, and poor therapeutic response. Existing evidence indicates that controlling lipid metabolism can be a potential strategy for cancer prevention and therapy. By reviewing the current literature on the lipid metabolism in various cancers, we summarized major lipid molecules including fatty acids and cholesterol as well as lipid droplets and discussed their critical roles in cancer cells and non-cancer in terms of either promoting- or anti-tumorigenesis. This review provides an overview of the lipid molecules in cellular entities and their tumor microenvironment, adding to the existing knowledge with lipid metabolic reprogramming in immune cells and cancer associated cells. Comprehensive understanding of the regulatory role of lipid metabolism in cellular entities and their tumor microenvironment will provide a new direction for further studies, in a shift away from conventional cancer research. Exploring the lipid-related signaling targets that drive or block cancer development may lead to development of novel anti-cancer strategies distinct from traditional approaches for cancer prevention and treatment.

## Introduction

Tumor cells are not isolated entities. In fact, the cellular microenvironment plays a critical role in carcinogenesis. For example, hypoxia and nutritional deficiency in the microenvironment usually contribute to a carcinogenic sequence [1, 2]. The crosstalk between tumor cells and the tumor microenvironment (TME) is considered by both basic scientists and clinicians as a pivotal feature of cancer that can be targeted for prevention and therapy [3]. In normal cells, a network system for the metabolism of lipids and glucose supports cell growth and function. In contrast to normal cells that use energy from respiration, most tumor cells depend primarily on fermentative glycolysis, referred to as the “Warburg effect”, even in the presence of oxygen [4]. Warburg effect is characterized by regeneration of NAD + from NADH in the pyruvate to lactate step that completes aerobic glycolysis. Warburg effect is not an efficient means of generating ATP, however the production of lactate from glucose metabolism through Warburg effect occurs 10–100 times faster than the complete oxidation of glucose in the mitochondria [5]. Therefore, Warburg effect is critical for the rapid biosynthesis to support cancer cell growth and proliferation, as a supply line not only generating ATP but also producing NADPH for lipid generation. The Warburg effect implications including growth advantages to cancer cells, chemo-resistant, and therapeutic targets have been reviewed previously [6–8], however biosynthetic programs of lipids in cancer cells are largely unknown. Further research is needed to elucidate the Warburg Effect functions in lipid biosynthesis in cancers. Lipid molecules, such as fatty acids, cholesterol, and lipid droplets, are used to provide energy and cell-synthetic materials for a variety of cellular processes. The process of lipid and glucose metabolism is also critical for maintaining tumor cell growth and function. However, in cancer cells, over-consumption of glucose, lipids, and amino acids is necessary to supply energy and cell-synthetic materials for pro-tumorigenic processes, including rapid proliferation, metastasis, and invasion of cancer cells [9, 10]. As lipid molecules provide not only the necessary materials but also energy for cancer cell growth, they may play key roles in carcinogenetic signaling during maladaptive transformation and development of tumor cells. In 1953, it was reported that tumor cells can synthesize lipids that are required for rapid tumor proliferation [11]. Since then, the role of lipid metabolism in carcinogenesis has evoked research interest. A large number of studies have demonstrated the correlation between abnormal lipid metabolism and various cancers, including breast cancer [12], prostate cancer [13], colon cancer [14], liver cancer [15], and etc. Thus, the connection between the lipid metabolism and the tumor microenvironment is highly significant. Increasing evidence points to several lipid signaling-associated biomarkers as potential diagnostic and therapeutic strategies to treat various cancers [16]. Cancer cells can also rewire lipid metabolism to mediate the development of acquired drug resistance [17], while targeting lipid metabolic enzymes (e.g., fatty acid synthase) re-sensitize breast cancer resistant to HER2-targeted therapies [18]. Several reviews have contributed greatly to the current knowledge on the roles of lipid metabolism, lipid biomarkers, diagnosis and therapeutic intervention, and drug resistance in cancers [19–23]. The approved cancer metabolic drugs and the small-molecule metabolic inhibitors in cancer clinical trials have been outlined and discussed in a recent review article [24]. In addition, lipid nanoparticles and lipid carriers are considered as a major research area for drug delivery and cancer cell targeting/killing, but this area is beyond the scope of the current review. This review, adding to the existing knowledge, focuses on the lipid-associated tumor microenvironment to discuss the roles of diverse types and multiple functions of lipid molecules in cancer and in the immune response to cancer.

## Tumor microenvironment

Accumulating evidence indicates the active role of TME played in carcinogenetic initiation, progression, metastasis, recurrence, and therapeutic responses. In addition to the cancer cells, TME is populated by many highly heterogeneous groups of non-cancer cells, including endothelial cells, adipose cells, immune/inflammatory cells, myeloid-derived suppressor cells (MDSCs), as well as other tumor-associated cells such as cancer-associated fibroblasts (CAFs), cancer-associated adipocytes (CAAs), tumor-associated neutrophils (TANs), tumor-associated macrophages (TAMs) [25–27]. TME has been widely accepted as an arena where the tumor cells undergo a metabolic remodeling to meet their needs for growth and survival to compete or cooperate with other non-cancer cells for nutrients and cell-signaling molecules [28]. When TME becomes hypoxic because vasculatures are inadequate, cancer cells can bypass the bloodstream to acquire nutrients and self-growth signaling molecules from TME [29]. In the extracellular space, the communication between the cancer cells and non-cancer cells is manifested by a complex network through the soluble factors such as inflammatory chemokines, cytokines, matrix remodeling enzymes, and various growth factors [28, 29]. For example, fibroblasts, embedded within the fibrillar extracellular matrix (ECM), can be activated by the ECM-degrading proteases, showing a phenotype of CAFs which promote tumorigenesis and trigger chemoresistance through a paracrine manner interacting with the adjacent epithelial tissues [30, 31]. It has also been highlighted that dynamic crosstalk between adipocytes and cancer cells is mainly sustained by the steroid hormones, adipokines, and cytokines, leading to the reprogramming of adipocytes to generate CAAs, which affect cancer cells during all steps of tumor progression by releasing adipokines, growth factors, and metabolites [32, 33]. Nowadays, numerous studies have indicated that tumor-associated immune/inflammatory cells promote and accelerate metastasis by establishing an immunosuppressive microenvironment within primary lesions and suppressing tumor immune surveillance [34]. Notably, the terminology of TANs does not relate to a specific differentiation step and activation status [35] but neutrophils as the first mediators of inflammatory reactions can influence tumor progression, depending on the tumor microenvironment [36]. A study indicates that TANs recruit macrophages and Treg cells, contributing to an immunosuppressive microenvironment to promote HCC progression and chemoresistance [37]. In parallel, there are also coexisted heterogeneous macrophage populations which influence both tumor growth and immune response in the tumor compartment [38]. M1-like macrophages are the subpopulation for anti-tumor immunity, whereas M2-like TAMs promote carcinogenetic progression and suppress immune response [36]. As the aberrant lipid metabolism can reprogram not only the cancer cells but also the surrounding non-cancer cells, TME is becoming very important research field to study lipid molecules contributing to cellular lipid metabolism reprogramming.

## Fatty acid

**Fig. 1:**
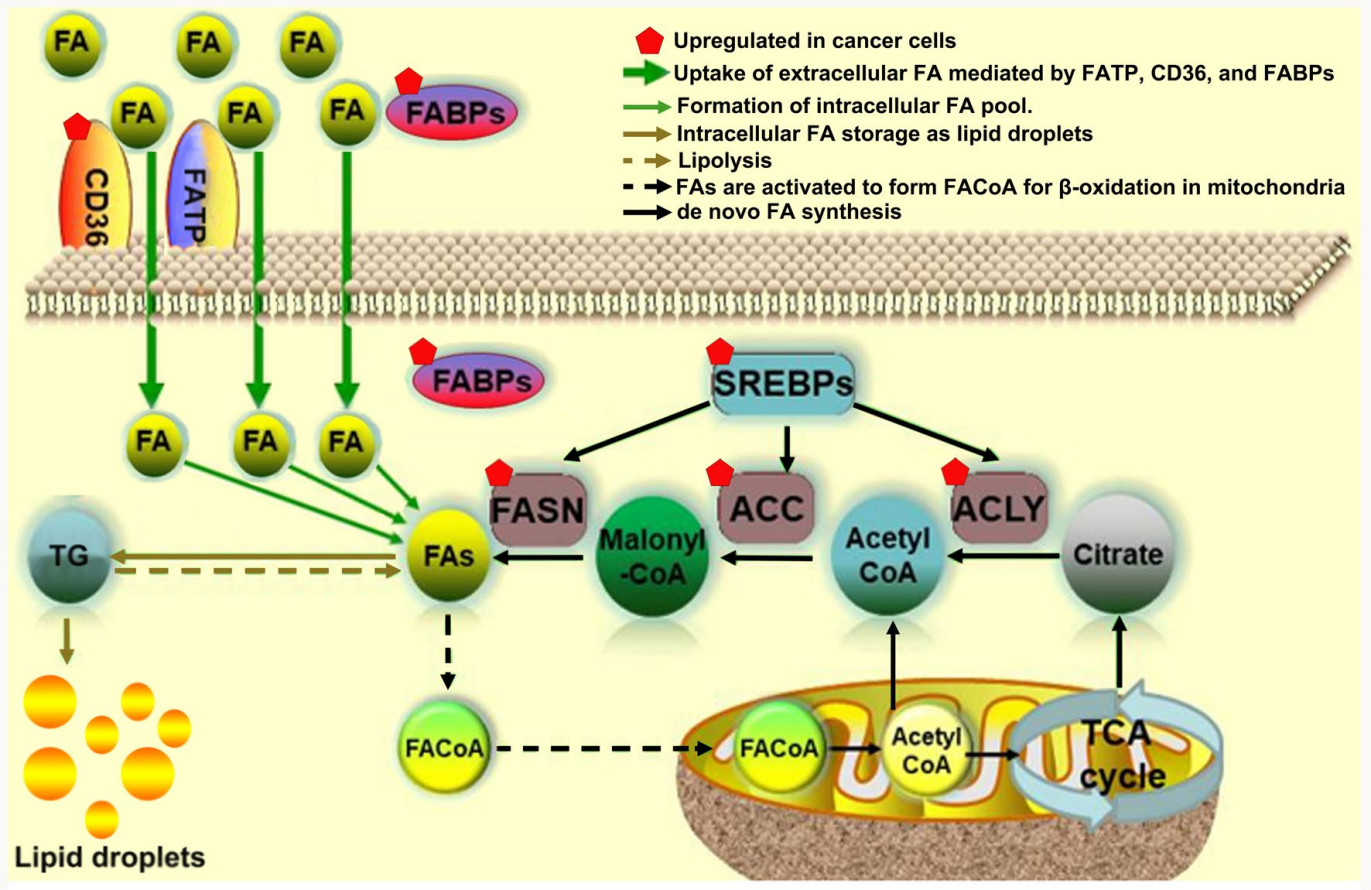
FA metabolic events and upregulated carcinogenetic signals. Scheme showing cellular fatty acid metabolic events, including FA transport, FA storage, lipolysis, β-oxidation, and de novo FA synthesis. Uptake of extracellular FAs is mediated by FA transport protein (FATP), CD36, and FABPs. The intracellular FAs form FA pool, and the FAs are either stored as lipids droplets or activated by reaction with CoA to form fatty-acyl-CoA (FACoA) for β-oxidation in mitochondria. The de novo FA synthesis begins with acetyl-CoA which is either derived from mitochondria or converted from citrate by ACLY. Acetyl-CoA is then converted to malonyl-CoA by ACC, and malonyl-CoA is further converted to FA by FASN. Red pentagon: upregulated FA metabolic signals contribute to carcinogenesis

Most mammalian cells obtain lipolytic free fatty acids (FFAs) from the blood. When fatty acids (FAs) enter proliferative fibroblast cells such as HeLa and H460 cells [39], they combine with fatty acid binding proteins (FABPs). Generally, FAs are transported to various cellular organelles by binding to the FABPs [40, 41]. Overexpression of FABPs has been reported to be significantly related to the malignant degree of breast cancer and poor prognosis in patients [12, 42]. FABPs are reported to play a key role in tumor initiation and progression in ovarian cancer and glioblastoma [43, 44]. Studies show that upregulation of FABP5 can activate peroxisome proliferator-activated receptor-β/δ (PPARβ/δ) and increase FABP5 methylation levels in CPG islands to accelerate the proliferation of cancer cells [45–47]. Tumor cells can also increase the uptake of FAs from plasma by upregulating cell-surface receptors (e.g., cluster of differentiation 36 [CD36] [48, 49]) to facilitate the transport of FAs. CD36 has been widely reported as a prognostic marker in various cancers, including gliomas and breast, prostate, ovary, colon, and liver cancers [50]. It has been shown that the suppression of CD36 successfully inhibits the metastasis of tumor cells, whereas overexpression of CD36 reverses this inhibitory effect. In this regard, CD36, as a key receptor for tumor metastasis, has been suggested as a therapeutic target [51, 52]. Most importantly, cells can produce FAs from citric acid by de novo synthesis via enzymatic reaction using adenosine triphosphate [ATP] citrate lyase (ACLY), acetyl coenzyme A carboxylase (ACC), and fatty acid synthase (FASN). De novo FA synthesis is accepted as a defining characteristic of cancer cells [53]. Upregulated expression of FASN significantly increases FA production by de novo synthesis, leading to a deteriorated outcome of breast cancer [54]. In contrast, the viability of cancer cells notably decreases when they are treated with FASN inhibitors such as triclosan and orlistat [55]. Similarly, upregulation of other enzymes such as ACLY and ACC has also been demonstrated to promote proliferation of cancer cells in glioblastoma, colorectal cancer, lung cancer, liver cancer, prostate cancer, and other tumors [56, 57]. The FA cellular metabolic events as well as the upregulated carcinogenetic signals during metabolic processes are summarized in Fig. 1. Of note, de novo lipogenesis is not the cause of malignancy and the tumor metabolism relate to the ability of FASN cannot elicit its malignant capabilities. Because FA synthesis expends energy, this is not an advantage for the survival of cancer cells. Additional work is required to fully understand the regulatory actions of FA de novo synthesis in cancer cells.

Epidemiological and experimental studies reveal the modulatory effects of FA on tumorigenesis [58, 59], while polyunsaturated fatty acids (PUFAs) are the extensively investigated FAs in various human cancer cells, e.g., colon cancer, glioblastoma, breast cancer, and prostate cancer. Previously, studies have demonstrated that gamma-linolenic acid, arachidonic acid, and eicosapentaenoic acid have selective tumoricidal actions by either augmentation of free-radical generation and lipid peroxidation or cytokine-mediated antitumor effects [60, 61]. As the precursors of prostaglandins, gammma-linolenic acid, dihomo-gamma-linolenic acid, and arachidonic acid are reported to not only prevent genetic damage but also augment immune responses and tumoricidal actions of macrophages [62]. Lately, accumulating evidence suggests that PUFAs suppress tumor growth by a variety of mechanisms depending on the cell types and the metabolism of PUFAs being handled by tumor cells. In human colon cancer cells, both n-3 PUFAs (α-linolenic acid, eicosapentaenoic acid and docosahexaenoic acid) and n-6 PUFAs (linoleic acid, gamma-linolenic acid and arachidonic acid) are demonstrated to trigger apoptosis through a mitochondrial pathway [63]. Supplementation of various n-3 PUFAs and n-6 PUFAs to human prostate cancer cells (RWPE-1 and PC-3) enhances the content of their long-chain metabolites and inhibits proliferation in these cells, but there was no correlation between inhibition of cell proliferation and free radical generation [64]. In human glioblastoma cell (T98G), arachidonic acid (n-6 PUFA) supplementation inhibits the growth of T98G cells though up-regulated level of arachidonoylethanolamide, a endogenous cannabinoid ligand, while eicosapentaenoic acid (n-3 PUFA) reduced the oleic acid (non-EFA) enhanced proliferation in breast cancer cells (MCF7) [65]. In mouse myeloma cells (SP2/0), alpha-linolenic and eicosapentaenoic acids induce suppression of SP2/0 cell proliferation which is dependent on the activities of cyclooxygenase, lipoxygenase, and superoxide [66]. The tumoricidal action of PUFAs (γ-linolenic acid, arachidonic acid and docosahexaenoic acid) is also found to associate with modulation of the expression of microRNAs and their targeted genes to trigger apoptosis in glioma cells [67]. In addition, studies suggest that PUFAs are capable to improve the therapeutic efficacy of chemotherapy on the drug-resistant cancer cells by enhancing drug uptake and reducing its efflux [68]. Exogenous FA is also reported to have tumoricidal action, for example, F-6 (a C-20 furanoic acid from Arabian Gulf catfish skin) can suppress proliferation and promote apoptotic cell death in leukemic and breast cancer cells [69]. Taken together, it is highly promising for FAs, particularly PUFAs, to be used as potential anti-cancer drug candidates for clinical patients.

## Cholesterol

**Fig. 2:**
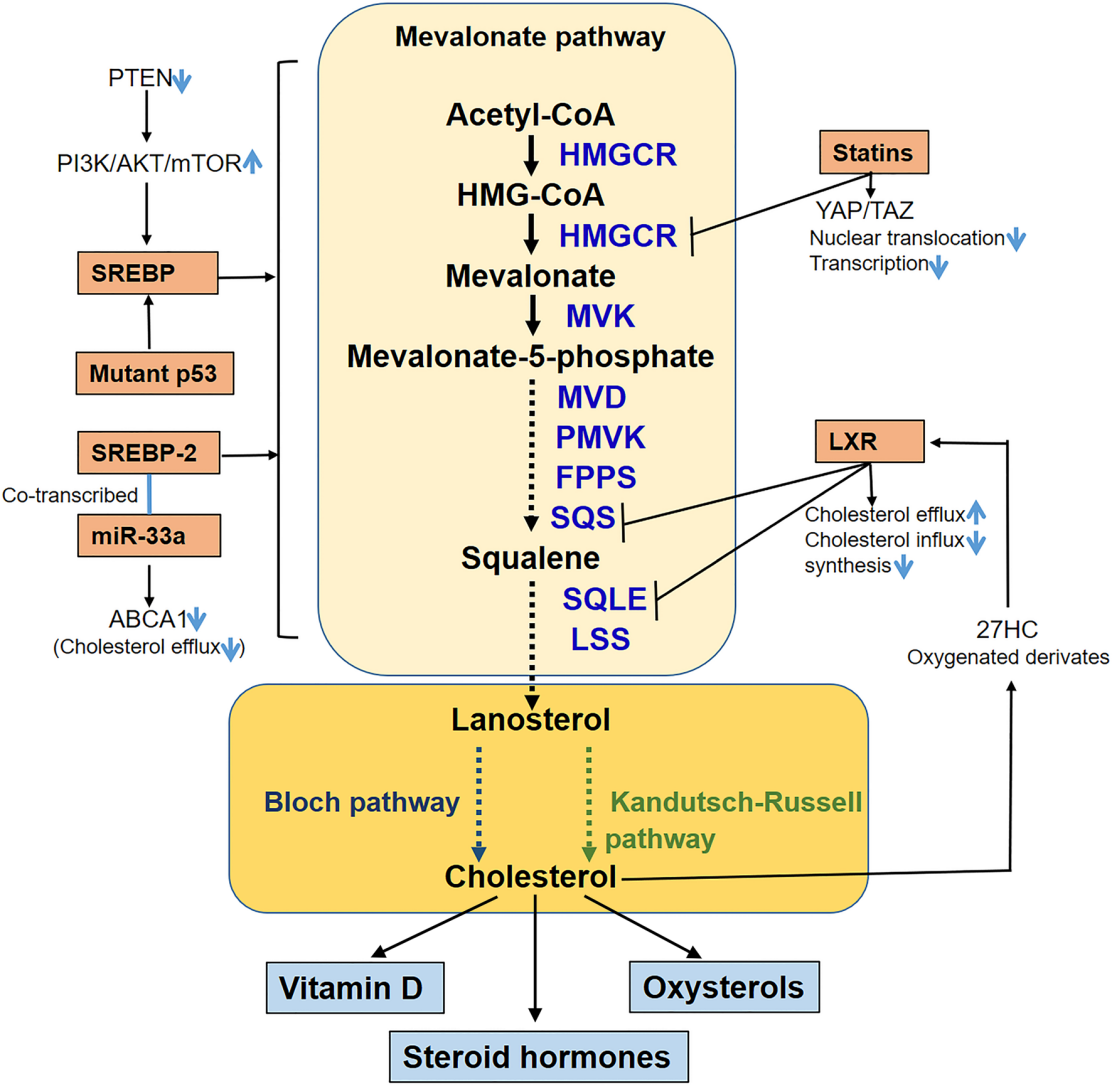
Pathways of cholesterol synthesis and regulation of carcinogenetic signals. Scheme showing cholesterol synthetic pathways, carcinogenetic signaling regulation in mevalonate pathway, and the cholesterol oxygenated metabolites feedback on cholesterol synthesis and efflux/influx. PI3K/AKT/mTOR and mutant p53 activate the mevalonate pathway via upregulation of SREBP, while Statins and LXR can inhibit cholesterol synthesis. 27HC negatively feedbacks on cholesterol synthesis and efflux/influx but miR-33a decreases cholesterol efflux. *MVK* mevalonate kinase; *MVD* mevalonate diphosphate decarboxylase; *PMVK* phosphomevalonate kinase; *FPPS* farnesylpyrophosphate synthase; *SQS* squalene synthase; *SQLE* squalene epoxidase; *LSS* lanosterol synthase

Cholesterol is an essential lipid molecule for the growth of all eukaryotic cells. The synthesis and metabolism of cholesterol are highly conserved in various organisms, from yeast to humans. Because de novo cholesterol synthesis is energetically expensive, most cells can take the premade cholesterol from circulating lipoproteins. As excessive cholesterol is harmful to the cell, cholesterol synthesis is therefore aimed at supplementing that exogenous supply based on demand, while elaborate mechanisms have evolved to tightly regulate cholesterol levels [70]. Cholesterol synthesis, in brief, begins with acetyl-coenzyme A derived from mitochondria and transported to the cytosol. In cytosol, one molecule of acetyl-coenzyme A and one molecule of acetoacetyl-CoA sare converted to HMG-CoA, being catalyzed by 3-hydroxy-3-methylglutaryl (HMG)-CoA synthase (HMGCS). The subsequent steps occur in the endoplasmic reticulum where HMG-CoA is reduced to mevalonate by HMG-CoA reductase (HMGCR). Mevalonate is phosphorylated to isopentyl pyrophosphate which is further converted to geranyl pyrophosphate. Two molecules of isopentyl pyrophosphate are condensed to form farnesyl pyrophosphate. Squalene synthase catalyzes the condensation of two molecules of farnesyl pyrophosphate to form squalene. Squalene is then cyclized to form lanosterol [71]. Subsequently, cholesterol is produced from lanosterol via the Bloch or Kandutsch-Russell pathway. Cholesterol synthesis is strictly controlled in normal cells, but not in tumor cells [72]. In fact, the pathways of cholesterol synthesis are closely associated with tumor development [73], for example, the mevalonate pathway has been reported to be a critical regulator of tumor progression and a therapeutic target [74]. Almost all the genes encoding cholesterol synthesis enzymes are transcribed through the regulation of sterol regulatory element-binding proteins (SREBPs), while the SREBP family of transcription factors is activated in response to low sterol status and helps coordinate the cholesterol synthesis [75]. SREBP-2 binds to the sterol regulatory elements in the promoters of genes such as HMGCR and mevalonate kinase (MVK) to activate and regulate the enzymes in mevalonate pathway, involving in the progression of various cancers [76]. Cholesterol- and steroid-dependent tumor cell proliferation has been widely reported [77]. MicroRNAs (miRNAs) have also been reported as transcriptional modulators of cholesterol metabolism and play a pivotal role in tumorigenesis [78, 79]. For example, miR-33a, an internal miRNA located in the gene encoding SREBF-2, mediates cholesterol metabolism and promotes tumor proliferation [80, 81]. Furthermore, multiple regulatory pathways for cholesterol signaling have been suggested as potential chemotherapy targets [82–84]. The metabolic intermediates of cholesterol are also major factors accounting for tumor initiation and progression [75]. Both cholesterol and metabolic intermediates for cholesterol are recognized to play a deteriorative role in various cancers. For example, 27-hydroxycholesterol (27HC), a cholesterol metabolite, is a selective estrogen receptor modulator [85]. In a murine breast cancer model, it was found that 27HC can accelerate carcinogenetic initiation and progression [85]. Alternatively, 27HC can activate the liver X receptor (LXR), which has a notable effect on malignancy [86, 87], e.g., promoting metastasis in pulmonary tumors [88]. The metabolic intermediates of cholesterol are also found to play a critical role in the emergence and development of prostate cancer [89, 90]. In addition, squalene monooxygenase, a rate-limiting enzyme in the metabolic process of cholesterol, has been reported to act as a reliable indicator of angiogenesis in prostate cancer [13]. The mevalonate pathway which is responsible for de novo cholesterol synthesis is significantly upregulated by mutant p53, while mutant p53 depletion phenotypically reverts breast cancer cells to a more acinar-like morphology, implicating down-regulation of cholesterol synthesis as a therapeutic target for tumors bearing mutations in p53 [91]. Targeting low-density lipoprotein receptor (LDLR) with LXR agonist causes inducible degrader of LDLR (IDOL)-mediated LDLR degradation and increased expression of the cholesterol efflux transporter to promote tumor cell death in glioblastoma [92]. On the other hand, the evidence is inconsistent in women; for example, cholesterol level is reported to be inversely related to the risk of gastric cancer among postmenopausal women [93]. Pathways of cholesterol synthesis and regulation of carcinogenetic signals are summarized in Fig. 2.

## Lipid droplets

Lipid droplets (LDs), independent organelles produced by the endoplasmic reticulum and Golgi apparatus to store excess lipids, are wrapped around double lipid components [94–98]. Most lipids stored in LDs are neutral lipids, such as FFAs and cholesterol. The gradual fusion of small LDs contributes to larger LDs, and the release of long-chain FAs (LCFAs) and cholesterol from LDs occurs through an enzymatic reaction [94, 98]. Multiple components exist in LDs, and these components may vary among different types of cells. Triglyceride (TG) is the main component in LDs found in fatty cells, while cholesteryl ester is mainly located in macrophage LDs [99]. The concept of LDs has improved our understanding of the potential carcinogenesis in terms of cancer cell adaptability and resilience to microenvironmental stress [100]. The changes in LD components correspond to alterations in the tumor cell phenotype and the surrounding microenvironment [101, 102]. LDs are important energy resources, and many organs obtain energy from LDs via FA oxidation and beta oxidation in mitochondria [103, 104]. In tumor cells, the LD energy supply can become the main source of energy for most cancers, including colon, prostate, ovarian, and breast cancer [105, 106]. LCFAs are supplied from circulation as part of TGs and reach various tissues, as well as tumor cells [107]. Free LCFAs can be directly taken up through binding to cellular lipoproteins, while the albumin-combined LCFAs can be transported into the cytoplasm through the FA transport molecule CD36 located on the surface of cells. Once inside the cell, LCFAs can diffuse into LDs [108, 109]. Subsequently, the LCFAs in LDs provide energy through beta oxidation and are reported to play a fundamental role in the pathogenesis of melanoma, ovarian cancer, and breast cancer [110]. The carcinogenetic transformation in clear cell renal cell carcinoma (ccRCC) is also related to abnormal lipid storage in LDs [111]. Increased accumulation of cholesterol, via hypoxia-inducible factor-1-dependent LDLR, further escalates the formation of LDs, contributing to the initiation of ccRCC [112]. In addition, SREBPs regulate, through sterol regulatory elements in gene regulatory region, the activation of several genes including FASN and stearoyl-CoA desaturase (SCD), which are the key factors governing the formation of LDs [111]. In parallel, SCD-1 is essential for ccRCC cell growth, whereas inhibition of SCD-1 induces apoptosis in ccRCC. A recent study demonstrated a key role of autophagy in LD formation during ccRCC pathogenesis. An autophagy-related protein, microtubule-associated protein 1S (MAP1S), suppresses ccRCC tumorigenesis by negatively regulating LD formation [113]. In prostate cancer, both cell proliferation and migration are positively correlated with LD formation [114, 115]. Prostate cancer can be divided into androgen-dependent and non-androgen-dependent subtypes. The expression of androgens is related to SREBP-1 expression [114]. According to Raman spectrum analysis, the number of LDs increases with the elevation of androgen levels [101]. LDs have also been reported to affect other hormone-dependent breast and ovarian cancer cells. The formation of LDs in breast cancer is correlated with estrogen receptor and progesterone receptor [116–118], while the development of ovarian cancer is controlled by increased LD formation, which is mediated by FASN expression [119, 120]. In addition to hormone-dependent cancer cells, LDs in non-hormone-dependent cancer cells also play a crucial role in tumor development. For example, the number of LDs in colon cancer cells is much higher than that in normal cells, and the elevated number of LDs show a direct regulatory effect on the growth of colon cancer cells [14, 121]. Recently, extracellular vesicles (EVs), phospholipid lipid bilayer particles with abundant cholesterol and ceramide, have attracted much attention. Moreover, EVs may play central roles in the metabolism and signaling pathways of the tumor microenvironment [122]. For examples, EVs, as cargos, can deliver the parental cells’ genomic and proteomic information to the surrounding/distant recipient cells to modulate their behavior [123]. EVs are also novel drug resistance modulators that add to the complexity of resistance mechanisms [124]. The use of EVs as novel cancer therapeutics has potential to improve clinical outcomes in patients [125].

## The role of lipid metabolism in the immune response to cancer

Growing evidence has highlighted the key role of lipid metabolism as a major influence in immune responses to cancer. As reported previously, T cell subsets have different metabolic traits that direct T cell survival, proliferation, and effector functions [126]. For example, increased fatty acid oxidation (FAO) level is found in activated CD4^+^ T cells but not in CD8^+^ T cells, which rapidly produce adenosine triphosphate via glycolysis for energy supply [127, 128]. Cancer cells can rewrite T cell metabolic programs to create a suppressive tumor microenvironment; for example, PD-1-expressing cancer cells alter the metabolic program of tumor-infiltrating T cells (TILs) by enhancing FAO level and inhibiting glycolysis [129]. Cancer cells can also impair T cell metabolism and effector functions by competing for key nutrients such as glucose and glutamine to drive tumor progression [130]. Furthermore, tumor-derived regulatory T (Treg) cells can interact with responder T cells to trigger cell senescence by rewriting their metabolic programs [131]. Treg cells can also modulate lipid metabolism in tumor-associated macrophages (TAMs) to promote tumor suppression [132]. TAMs represent a subpopulation that acquires an M2-like tumor-promoting phenotype characterized by high levels of immunosuppressive markers such as Arginase 1 (ARG1) and Interleukin (IL)10 [133]. In fact, lipid metabolic traits of macrophages are reflected by their M1/M2 polarization state, a classical (M1) or alternative (M2) activation in response to microenvironmental signals, showing either anti- or pro-tumorigenic properties [134]. In macrophages, lipid metabolism is oriented toward fatty acid synthesis (FAS) in M1 polarization, while M2 polarization activates FAO to generate ATP and produce acetyl-CoA, which participates in the Krebs cycle (TCA cycle) and cholesterol biosynthesis [135]. FFAs can stimulate tissue-resident macrophages to release cytokines, which tune hematopoiesis to further engage immune cells into tissues [136]. Cancer cells drive the cells, represented by TAMs as well as MDSCs. In tumor-infiltrating MDSCs, the use of energy is shifted from glycolysis toward FAO, which is characterized by CD36-mediated fatty acid uptake and upregulated expression of FAO enzymes [137]. In addition, increased levels of FAS and intracellular TG storage are also found in tumor-associated dendritic cells [138, 139]. Modulating the key lipid molecular players of lipid metabolism appears to be a promising tool for tuning immune responses to boost the intrinsic anti-tumor activity of both adaptive and innate immune compartments.

**Fig. 3:**
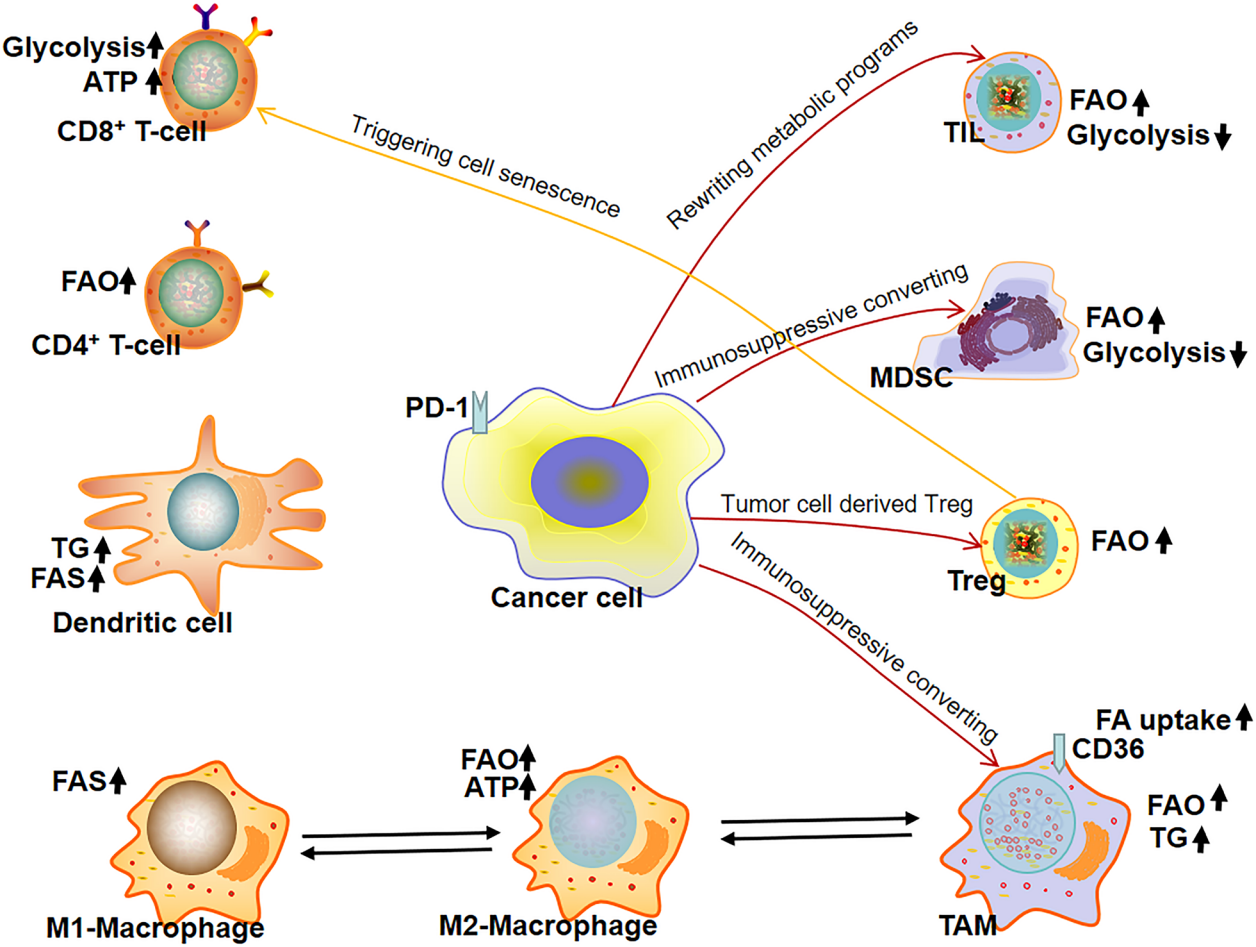
Lipid metabolism of immune cells and the immunosuppressive cells in tumor microenvironment. Scheme showing lipid and energy metabolism of immune cells and the immunosuppressive cells in tumor microenvironment. The tumor suppressive immune cells with warm-toned in cytosol are shown in left, while the tumor-promoting cells with cold-toned in cytosol are shown in right. Increased fatty acid synthesis in dendritic cells and increased fatty acid oxidation in CD4^+^ T cells are found in tumor microenvironment. The responder CD8^+^ T cells (cytotoxic T cells) produce ATP via glycolysis for energy supply, however, tumor cell can rewrite the lipid metabolic programs of TILs which decrease glycolysis but increase FAO for energy supply. Tumor cell can also concerting MDSCs and TAMs to immunosuppressive cells. Treg cells can trigger senescence of cytotoxic T cells. Increased FAO is also found in the immunosuppressive cells (MDSC, Treg, and TAM) as well as in M2-macrophage

Regarding the lipid molecules, FAs have been reported to interfere with immune responses such as the modulation of lymphocyte proliferation and natural killer activity [140, 141]. A recent review article discussed the regulatory roles of the fatty acid palmitate in controlling immune balance, in which palmitate could modulate innate immunity by not only regulating the activation of pattern recognition receptors in local innate immune cells but also coordinating the immunological activity in tissues [142]. High-fiber diets are associated with a decreased risk of colorectal cancer, but the anti-cancer mechanism is largely unknown [143, 144]. Studies have shown that short-chain fatty acids, the major microbial-derived metabolites, can modulate immune response through free fatty acid receptor 2 (FFAR2) which is highly expressed on immune cells, including myeloid cell populations [145] and Tregs [146]. In addition, FFAR2 can regulate macrophage cytokine expression [147], while dendritic cells from FFAR2-deficient mice are unable to promote promoted B-cell IgA production, resulting in unresolved inflammation and broken intestinal homeostasis [148]. Fatty acid-binding protein 5, a cellular chaperone of long-chain fatty acids being well-studied in various immune cells [149], is reported to regulate the commitment of dendritic cells and generation of Tregs in tumor microenvironment [150]. Cholesterol is also a key player for immune responses, e.g., the ATP-binding cassette transporter G1-dependent cholesterol efflux can suppress Tregs development [151]. The oxidized LDLs are found to be the main types of lipids accumulating in tumor-infiltrating lymphocytes [152], as a result of the immunosuppressive tumor microenvironment. For example, the CD36-mediated bad cholesterol uptake in CD8^+^ T lymphocytes can lead to their dysfunction and cancer progression [153]. Bioactive lipids are also played key roles in modulation of immune check point inhibitors, which has been discussed in a recent review article [154]. Therefore, comprehensively exploring the lipid metabolic profiles of immune cells and cancer cells will facilitate the development of novel strategies for cancer therapy. Lipid metabolism of immune cells and the immunosuppressive cells in tumor microenvironment is summarized in Fig. 3.

## Contribution of the current work to the existing knowledge

In comparison with other review articles, the current work adds to the existing knowledge in the following three aspects. (1) The canonical rationale for investigating lipid metabolism in cancer cells is to study the lipid requirements for plasma membrane synthesis and energy production. The current work emphasizes that abnormal lipid metabolism in cancer cells contributing to carcinogenic signaling within the tumor microenvironment. (2) The introduction of lipid metabolism has led to a paradigm change in the treatment of cancer. However, several challenges remain in exploring the carcinogenic roles of lipid molecules. In the current work, we summarize that the lipid molecules exhibit not only a tumorigenic effect but also an anti-tumorigenic effect, in the context of different cancer types; such contrasting effects merit further study. (3) We discuss the available studies on lipid metabolism in the immune response to cancer and suggest that key lipid molecular players can be promising tools for tuning such immune responses. Lipid metabolic reprogramming may help immune checkpoints restore effective anti-tumor immunity. Unlike previous studies that mostly focused on lipid metabolism in maintaining cellular structure and providing energy, the current work discusses the available studies on lipid metabolism related signaling targets in the tumor microenvironment that either promote or inhibit carcinogenesis. Exploring the lipid-related signaling targets that drive or block cancer development will provide a new direction for further studies, in a shift away from conventional cancer research. The limitations of the current work are as follows: (1) relationship between lipid molecules and oncogenes is not well discussed. There are functional links between lipid molecules and oncogenes. For examples, the growth factor receptor-driven cancers depend on membrane lipid remodeling for transduction of oncogenic signals [155]. Oncogene KRAS activates lipogenesis resulting in distinct proteomic and lipid signatures in lung adenocarcinoma [156]; (2) lipid signaling-based biomarkers for cancer diagnosis and treatment response are not discussed. Lipid molecules may be used as tumor-related biomarkers for diagnosis and treatment response; for example, positron emission tomography has been applied to observe the synthesis of tumor-active lipids using tracers [157]. Thus, further study is needed to explore these aspects.

## Conclusion

Lipid molecules, LDs, and metabolic intermediates for lipid biosynthesis are extremely critical for signaling between cancer cells and their surrounding microenvironment. Changes in lipid metabolism in cancer cells as well as in immune cells can affect initiation and progression of cancer in a subtle way. The proliferation of cancer cells depends on not only the energy and nutrient supplies, but also on the communication with their microenvironment, in which lipids inevitably function as prominent modulators. Comprehensively understanding the regulatory role of lipid metabolism in carcinogenesis would provide a novel strategy for cancer prevention and treatment.

## Data Availability

Not applicable.

## Abbreviations

FFAs: Free fatty acids
FAs: Fatty acids
FATP: FA transport protein
FABPs: Fatty acid binding proteins
CD36: Cluster of differentiation 36
ATP: Adenosine triphosphate
FACoA: Fatty-acyl-CoA
ACLY: ATP citrate lyase
ACC: Acetyl coenzyme A carboxylase
FASN: Fatty acid synthase
MVK: Mevalonate kinase
MVD: Mevalonate diphosphate decarboxylase
PMVK: Phosphomevalonate kinase
FPPS: Farnesylpyrophosphate synthase
SQS: Squalene synthase
SQLE: Squalene epoxidase
LSS: Lanosterol synthase
HMGCS: 3-Hydroxy-3-methylglutaryl (HMG)-CoA synthase
HMGCR: The enzyme 3-hydroxy-3-methylglutaryl-CoA reductase
SREBPs: Sterol regulatory element-binding proteins
27HC: 27-Hydroxycholesterol
miRNAs: MicroRNAs
LXR: Liver X receptor
LDLR: Low-density lipoprotein receptor
IDOL: Inducible degrader of LDLR
LCFAs: Long-chain FAs
ccRCC: Clear cell renal cell carcinoma
TG: Triglyceride
SCD: Stearoyl-CoA desaturase
MAP1S: Microtubule-associated protein 1S
EVs: Extracellular vesicles
FAO: Fatty acid oxidation
Treg: Regulatory T cell
TME: Tumor microenvironment
CAFs: Cancer-associated fibroblasts
CAAs: Cancer-associated adipocytes
TANs: Tumor-associated neutrophils
ECM: Extracellular matrix
TAMs: Tumor-associated macrophages
ARG1: Arginase 1
IL: Interleukin
FAS: Fatty acid synthesis
MDSCs: Myeloid-derived suppressor cells
FFAR2: Free fatty acid receptor 2
